# Intraventricular Sialidase Administration Enhances GM1 Ganglioside Expression and Is Partially Neuroprotective in a Mouse Model of Parkinson’s Disease

**DOI:** 10.1371/journal.pone.0143351

**Published:** 2015-12-02

**Authors:** Jay S. Schneider, Thomas N. Seyfried, Hyo-S. Choi, Sarah K. Kidd

**Affiliations:** 1 Department of Pathology, Anatomy and Cell Biology, Thomas Jefferson University, Philadelphia, PA, United States of America; 2 Department of Biology, Boston College, Chestnut Hill, MA, United States of America; St. Jude Children's Research Hospital, UNITED STATES

## Abstract

**Background:**

Preclinical and clinical studies have previously shown that systemic administration of GM1 ganglioside has neuroprotective and neurorestorative properties in Parkinson’s disease (PD) models and in PD patients. However, the clinical development of GM1 for PD has been hampered by its animal origin (GM1 used in previous studies was extracted from bovine brains), limited bioavailability, and limited blood brain barrier penetrance following systemic administration.

**Objective:**

To assess an alternative therapeutic approach to systemic administration of brain-derived GM1 to enhance GM1 levels in the brain via enzymatic conversion of polysialogangliosides into GM1 and to assess the neuroprotective potential of this approach.

**Methods:**

We used sialidase from *Vibrio cholerae* (VCS) to convert GD1a, GD1b and GT1b gangliosides to GM1. VCS was infused by osmotic minipump into the dorsal third ventricle in mice over a 4-week period. After the first week of infusion, animals received MPTP injections (20 mg/kg, s.c., twice daily, 4 hours apart, for 5 consecutive days) and were euthanized 2 weeks after the last injection.

**Results:**

VCS infusion resulted in the expected change in ganglioside expression with a significant increase in GM1 levels. VCS-treated animals showed significant sparing of striatal dopamine (DA) levels and substantia nigra DA neurons following MPTP administration, with the extent of sparing of DA neurons similar to that achieved with systemic GM1 administration.

**Conclusion:**

The results suggest that enzymatic conversion of polysialogangliosides to GM1 may be a viable treatment strategy for increasing GM1 levels in the brain and exerting a neuroprotective effect on the damaged nigrostriatal DA system.

## Introduction

Parkinson’s disease (PD) is a progressive neurodegenerative disorder primarily characterized by the loss of substantia nigra (SN) dopaminergic neurons and depletion of striatal dopamine (DA). Although there are effective treatments to lessen the signs and symptoms of PD, no therapy has yet been found to unequivocally slow the progression of the disease. Numerous preclinical studies though have shown that administration of GM1 ganglioside, a major component of plasma membrane lipid raft signaling domains, results in significant biochemical and behavioral recovery following different types of nervous system lesions [1,2], including those in animal models of PD. GM1 administration rescued damaged SN DA neurons, increased striatal DA levels and enhanced DA synthetic capacity in residual DA neurons in various animal models of PD [3–10].

Positive preclinical results with GM1 in mouse and non-human primate MPTP models of PD have translated to positive clinical data. In a 16 week double-blind placebo controlled study, a mild symptomatic effect was detected in GM1-treated subjects (vs. placebo-treated subjects) on measures of motor function [11]. A follow-up open extension of that study found that long-term (i.e., five years) use of GM1 resulted in modest symptom progression (compared to expected symptom progression) and a number of subjects had lower (improved) motor function scores after five years of GM1 use than they had at baseline prior to randomization into the original study [12]. More recently, a double-blind placebo controlled delayed start study of GM1 in PD reported that GM1 had an early-appearing symptomatic effect (similar to that previously described) and significantly slowed symptom progression over a 2 year period [13]. An imaging sub-study of the larger delayed start study examined effects of GM1 on dopamine transporter binding, as a surrogate measure of disease progression, and reported slowing of loss of binding potential (BP_ND_) values in several striatal regions in GM1-treated subjects and in some cases, an increased BP_ND_ in some striatal regions was detected after GM1 use [14].

Although these data suggest that GM1 may have neuroprotective/neurorestorative effects in PD, its clinical development has been hampered by its animal origin (GM1 used in previous studies was extracted from bovine brains), limited bioavailability, and limited blood brain barrier penetrance following systemic administration. An alternative therapeutic approach to systemic administration of brain-derived GM1 might be to enhance endogenous levels of GM1 *in vivo* in the brain. One approach to enhancing GM1 levels involves the manipulation of ganglioside degradation by sialidases. The more highly expressed gangliosides in adult mammalian brain are GM1, GD1a, GD1b, GT1b, GQ1b, and to a much lesser extent GD3. GM1 is suggested to be broadly neuroprotective and based on *in vitro* studies. GD3, a minor ganglioside in adult mammalian brain, has been suggested to be a potential mediator of cell death [15,16], although this has not been confirmed *in vivo*. Sialidases hydrolyze sialic acid linkages on gangliosides and can degrade polysialogangliosides (and GD3) while increasing GM1 [17]. Dhanushkodi and McDonald [17] previously showed that brain ganglioside profiles can be altered in vivo by intraventricular infusion of *Vibrio cholera* (VCS) sialidase and that this protects against excitotoxic neurodegeneration. Yang et al. [18] also showed that infusion of sialidase from *Clostridium perfringens* (CPS) enhanced spinal axon outgrowth into implanted peripheral nerve grafts in a rat model of brachial plexus avulsion.

The present study assessed the extent to which administration of VCS directly to brain could increase GM1 levels and exert a neuroprotective effect similar to that observed with systemic administration of GM1 in a mouse MPTP model of PD.

## Material and Methods

### Animals, Surgical Procedures, and MPTP Administration

All animal procedures were approved by the Thomas Jefferson University Institutional Animal Care and Use Committee. Male C57Bl6 mice (age 7–10 weeks, Taconic Biosciences, Germantown, NY) were housed on a 12 hour light/dark cycle and had free access to food and water throughout the study. *Vibrio cholera* (VCS) sialidase (Prozyme, Hayward, CA) was prepared in sterile artificial cerebral spinal fluid (aCSF: 150mM Na, 3mM K, 1.4mM Ca, 0.8mM Mg, 1mM P and 155mM Cl) at doses of 0.25, 0.50, or 1.0 U/mL and loaded into Alzet osmotic minipumps (Model 2004, DURECT Corp., Cupertino, CA) according to the manufacturer’s instructions. These pumps deliver their contents at a constant rate of 0.25 μl/hour for 4 weeks. Control pumps containing only vehicle (aCSF) were also prepared. Brain infusion cannulae (ALZET Brain Infusion Kit 3, DURECT Corp., Cupertino, CA) were attached to the pumps, which were assembled approximately 24 hours prior to surgical implantation and primed in sterile saline at 37°C to ensure immediate delivery of sialidase or vehicle following implantation. On the day of surgery, animals were anesthetized with ketamine/xylazine (100/10 mg/kg, i.p.) and placed into a stereotaxic head holder. The skull was exposed and a subcutaneous intrascapular pouch was created in which to place the pump. The cannula was inserted into the third ventricle using the following coordinates: -0.22mm from Bregma, 0.87mm lateral to the midline, and 2.5mm below the cortical surface [19]. Post-surgery analgesia was achieved with buprenorphine (2.5 mg/kg, i.p.).

One week after surgery, animals received administration of MPTP-HCl (20 mg/kg, s.c., twice daily, 4 hours apart, for 5 consecutive days, Sequoia Research Products LTD, Berkshire, UK) and were euthanized 2 weeks after the final MPTP injection. Pumps were retrieved to verify that that the appropriate amount of fluid was administered to the animal. Additional animals (N **=** 16) received MPTP as described above and beginning 24 hours after the last MPTP injection, were administered GM1 ganglioside (N = 8, 30 mg/kg, i.p., Fidia Pharmaceutical Corp, Abano Terme, Italy) or saline (N = 8) once daily for 14 consecutive days. At the time of euthanasia, brains were removed quickly and the striatum contralateral to the side of infusion was dissected and frozen for later analysis. The remainder of the brain was submersion fixed in 4% paraformaldehyde for later histological processing.

### Measurement of Striatal Dopamine and Metabolite Levels

Striatal samples were sonicated in 0.4 M perchloric acid and centrifuged at 15,000 rpm for 5 min at 4°C. The supernatant was used for high-performance liquid chromatography (HPLC) analysis of DA and metabolites using a Coulochem III LC System (Thermo/Dionex, US), as previously described [20]. Peak heights were compared with internal standard values to determine the concentration of DA and metabolites (EZchrome V3.1, Agilent Technologies, Santa Clara, CA).

### Immunohistochemistry and Stereological Cell Counting

Fixed tissue blocks were immersed in 30% sucrose for cryoprotection and sectioned frozen on a sliding microtome (30μm section thickness). All sections through the rostro-caudal extent of the substantia nigra pars compacta (SNc) were collected and every third section was processed for tyrosine hydroxylase (TH) immunohistochemistry, with adjacent sections stained with cresyl violet [20]. Briefly, sections were washed in PBS and endogenous peroxidase activity was quenched using peroxide/PBS containing 0.3% Triton X-100. Sections were then blocked in 5% non-fat milk, washed, and incubated overnight with primary antibody (rabbit anti TH, 1:1,000, Pel-Freez, Rogers, AR). Following additional washes, sections were incubated in biotinylated secondary antibody, avidin biotin complex (VECTASTAIN Elite ABC system, VECTOR Laboratories, Burlingame, CA) and reaction product was visualized with NovaRED (VECTOR Laboratories, Burlingame, CA). Sections were then mounted on slides, dehydrated, cleared, and coverslipped.

Slides were coded and analyzed blindly to estimate the number of TH^+^ and cresyl violet stained cells in the region of interest (SNc) on one side in each animal by unbiased stereological counting (StereoInvestigator, MBF Bioscience, Williston, VT), as described previously [20]. The SNc was identified and outlined at 4x magnification and cells were counted at 100x magnification. Only cells with clear cytoplasmic staining and a distinct nucleus contained within the counting frame were counted. The same counting regions from an individual animal were used for both TH and cresyl violet counts. Cell counts were only accepted with a Gundersen CE of <0.1.

Sections from the striatum were used for visualization of gangliosides by fluorescence immunohistochemistry. Sections were washed in PBS and then blocked for 1hour at room temperature and incubated in primary antibody over night (rabbit anti-GM1 1:250 (Abcam, Cambridge, MA); mouse anti-GT1b 1:250 (EMD Millipore, Billerica, MA); mouse anti-GD1a 1:250 (Millipore, Billerica, MA); mouse anti-GD3 1:100 (Thermo Scientific, US)). Sections were then washed and incubated with biotinylated secondary antibody directed against the appropriate host (1:500) for 2 hours followed by treatment with ABC- horseradish peroxidase (HRP) reagent for 1hour. Signal detection was enhanced by further incubation with a fluorophore-conjugated anti-HRP antibody (Alexa Fluor 488 anti-HRP 1:500, Jackson ImmunoResearch Laboratories, Inc., West Grove, PA, or DyLight 488, Vector Laboratories, Burlingame, CA).

### Ganglioside Isolation, Purification and Quantification

Gangliosides were isolated and purified from lyophilized tissues using modifications of previously described procedures [21–23]. Samples were centrifuged at 2500 rpm for 20 min, and the supernatant was collected. The pellet and stirring bar were washed in 2 ml CHCl_3_: CH_3_OH 1:1 (v/v), centrifuged again and the second supernatant was combined with the first. The supernatant was brought up to 19.6 ml at a ratio of 30:60:8 CHCl_3_: CH_3_OH: dH_2_O (v/v/v). Neutral lipids were separated from acidic lipids and gangliosides using DEAE Sephadex (A-25, GE Healthcare, Upsala, Sweden) column chromatography procedure previously described [24]. Gangliosides were separated from the acidic lipids using Folch partitioning, base treated, and desalted as previously described [25].

Total ganglioside content was quantified before desalting using the resorcinol assay as described previously [25]. The resorcinol assay aliquot was estimated to contain 1.0 μg ganglioside sialic acid. The resorcinol assay was not performed after desalting as the preliminary isolation using mouse brain standard of similar weight yielded a low recovery rate (30%, not shown).

Gangliosides were analyzed qualitatively by high-performance thin-layer chromatography (HPTLC) according to modifications of previously described methods [25]. Gangliosides were spotted in 7-mm bands on the plate using a TLC spotter (CAMAG Scientific, Inc., Wilmington, NC). The amount of lipid spotted per lane was estimated to be 1.5 μg assuming a 30% recovery after desalting. The percent distribution of each band was determined using previously described methods [26]. The total ganglioside distribution was normalized to 100%.

### DA Uptake Assay

An additional set of animals received intraventricular infusion of sialidase (N **=** 3, VCS 0.5 U/ml) or aCSF only (N **=** 4) to examine potential effects of sialidase administration on DA uptake during the same period of time in which animals received MPTP during the main study. Thus, sialidase was administered for 12 days, encompassing a period equivalent to the 1week of sialidase infusion prior to MPTP administration and the 5 day MPTP exposure period. These animals were then euthanized to examine effects of sialidase treatment on DA transporter activity. Dopamine uptake was estimated in whole striatal homogenates as described previously [27]. Total uptake was measured after adding 100 **μ**l of fresh striatal homogenate to tubes containing assay buffer (50 mM Tris–HCl, pH 7.4; 120 mMNaCl; 5mMKCl; 0.32 M sucrose), 100 **μ**M pargyline and 0.5 **μ**M [^3^H]DA (specific activity = 78.0 Ci/mmol, Perkin Elmer, Waltham, MA). Nonspecific uptake was assessed by the addition of 100 **μ**M mazindol to the uptake assay buffer. The reaction proceeded at 37°C for 3 min, was terminated by placing the tubes in ice, and samples were quickly vacuum filtered onto Whatman GF/B filters using a Brandel Harvester (Brandel, Inc., Gaithersberg, MD). Following rinses with cold assay buffer, filters were dried, placed in scintillation vials containing 10 ml of scintillation fluid and radioactivity was quantified by liquid scintillation spectrometry. All samples were run in duplicate and results were normalized to protein concentration.

### Data Analysis

All data shown are expressed as means ± standard errors. Where appropriate a one-way analysis of variance (ANOVA) with Bonferroni or Dunnet’s post hoc multiple comparisons post hoc tests was used to determine statistical significance (defined as p < 0.05).

## Results

### Sialidase treatment partially protects against striatal dopamine depletion in MPTP-treated mice

**Fig 1:**
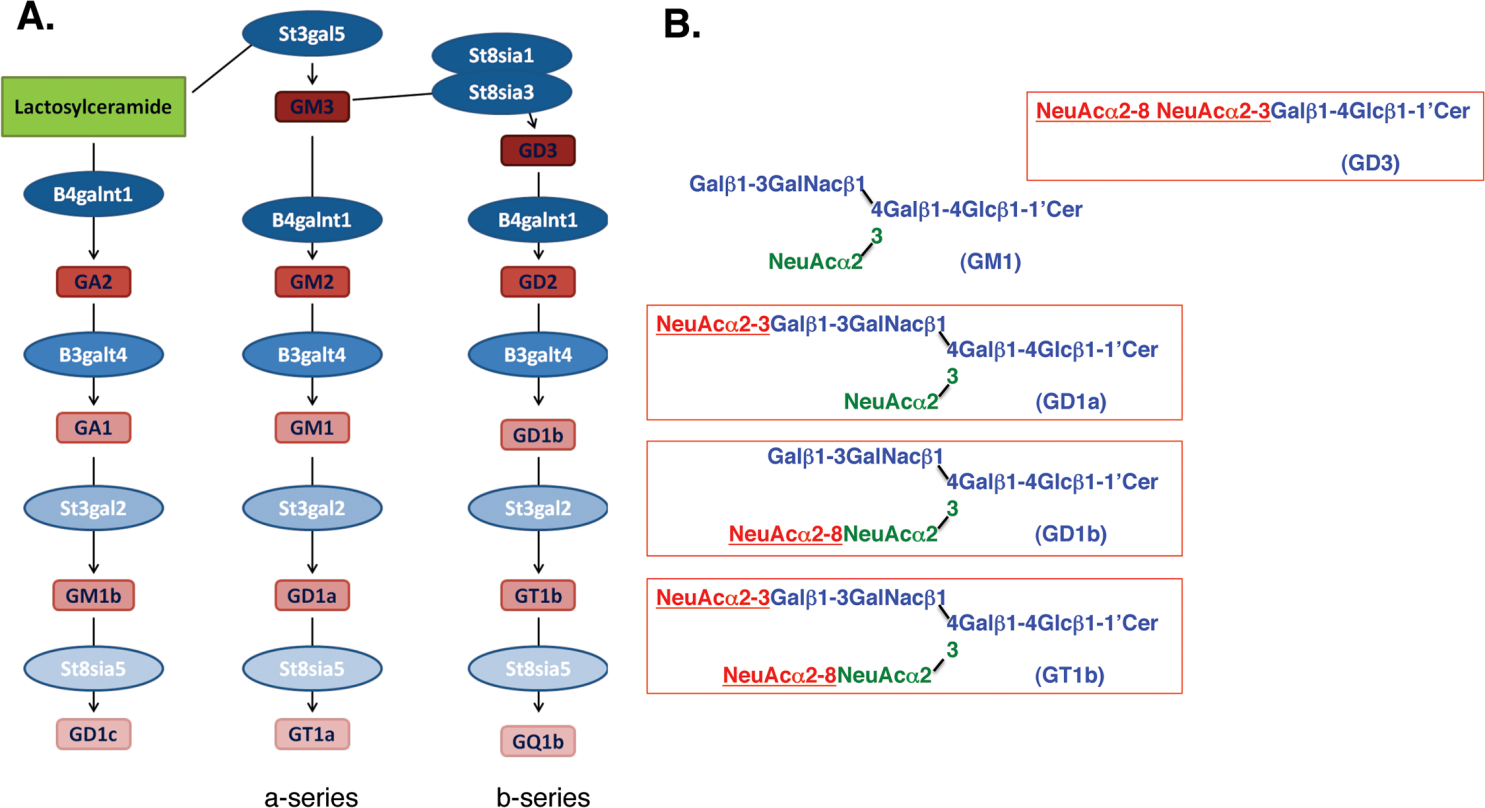
Ganglioside biosynthetic pathways and effects of *Vibrio cholerae* sialidase (VCS). A. The main gangliosides found in brain are from the a-series and b-series gangliosides. Gangliosides are synthesized by sequential addition of sugars and sialic acid residues to a sphingosine backbone through the action of various glycosyltransferases (ex., St3gal5, B4galnt1, B3galt4, St8sia1/St8sia3) and sialyltransferases (St3gal2, St8sia5). B. VCS hydrolyzes α2–8 and terminal α 2–3 sialic acid linkages (red) while internal α 2–3 linkages (green) are unaffected by VCS. Thus GD1a, GD1b, and GT1b, are converted to GM1 and the potentially apoptogenic ganglioside GD3 is degraded.

**Fig 2:**
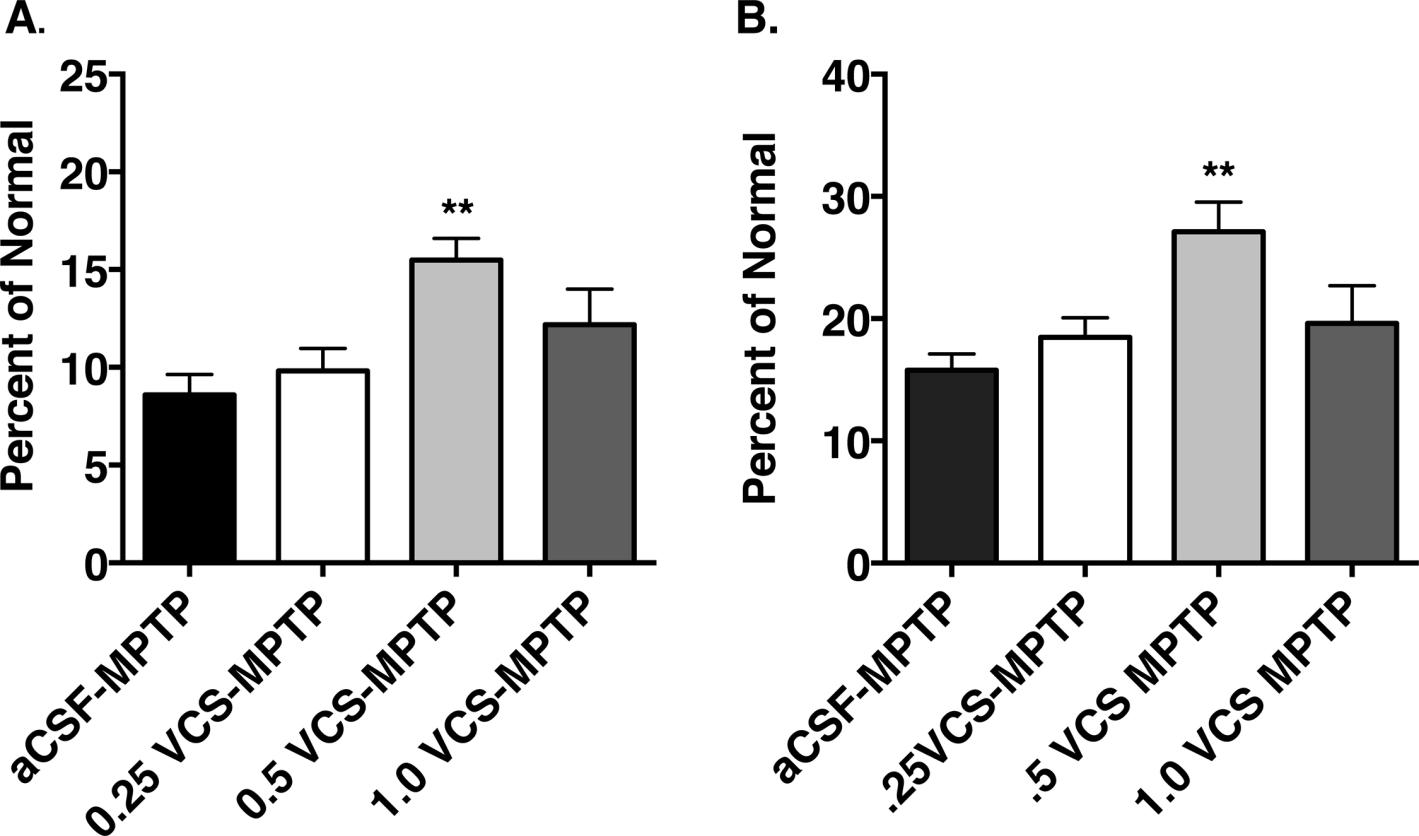
Effects of sialidase treatment on striatal dopamine and DOPAC levels. Sub-acute MPTP treatment (aCSF-MPTP) resulted in significant decreases in striatal dopamine (A.) and DOPAC (B.) levels. Intraventricular infusion of VCS at the 0.5 U dose partially protected striatal DA and DOPAC levels (**p<0.01 vs aCSF-MPTP).

Ganglioside biosynthetic pathways and the effects of VCS on ganglioside biosynthesis are depicted in Fig 1. Two weeks following the last MPTP exposure, striatal DA levels in animals that received intraventricular infusion of aCSF alone were reduced by 89.8 ± 1.3%. Infusion of VCS for 2 weeks resulted in a dose-dependent sparing of striatal DA levels (F_(3,23)_ = 5.36; *P* = 0.0060). The 0.25U and 1.0U doses did not have a statistically significant effect on striatal DA levels but the 0.5U dose significantly increased striatal DA levels compared to a CSF-treated animals (*t* = 3.72, *P* < 0.01) (Fig 2, Table 1). Administration of VCS to normal animals had no significant effects on striatal DA levels.

**Table 1:** Effects of sialidase and GM1 ganglioside administration on striatal dopamine (DA) and dihydroxyphenylacetic acid (DOPAC) levels.

Treatment	*n*	DA (μg/g wet tissue)	DOPAC (μg/g wet tissue)	DOPAC/DA
Normal/Saline	10	9.04 ± 0.39	0.88 ± 0.05	0.100 ± 0.008
Normal/GM1	8	8.27 ± 0.32	0.76 ± 0.03	0.092 ± 0.004
MPTP/Saline	8	0.87 ± 0.11^a^	0.21 ± 0.02 ^a^	0.252 ± 0.022
MPTP/GM1	8	2.64 ± 0.08 ^b^	0.35 ± 0.02 ^e^	0.134 ± 0.010 ^b^
Normal/aCSF	7	9.28 ± 0.55	0.81 ± 0.03	0.093 ± 0.004
Normal/0.25U/ml VCS	8	10.05 ± 0.56	0.88 ± 0.06	0.088 ± 0.005
Normal/0.50U/ml VCS	7	9.37 ± 0.47	0.92 ± 0.13	0.096 ± 0.011
Normal/1.0U/ml VCS	7	9.95 ± 0.54	0.99 ± 0.18	0.100 ± 0.150
MPTP/aCSF	9	0.77 ± 0.09 ^c^	0.15 ± 0.01 ^f^	0.173 ± 0.010 ^f^
MPTP/0.25U/ml VCS	8	0.88 ± 0.10	0.15 ± 0.01	0.180 ± 0.013
MPTP/0.50U/ml VCS	6	1.38 ± 0.10 ^d^	0.22 ± 0.02 ^g^	0.163 ± 0.013
MPTP/1.0U/ml VCS	5	1.09 ± 0.16	0.16 ± 0.03	0.142 ± 0.006

^a^ P < 0.0001 vs. Normal/Saline

^b^ P < 0.001 vs. MPTP/Saline

^c^ P < 0.001 vs. Normal/aCSF

^d^ P < 0.01 vs. MPTP/aCSF

^e^ P < 0.05 vs. MPTP/Saline

^f^ P < 0.0001 vs. Normal/aCSF

^g^ P < 0.05 vs. MPTP/aCSF.

Data shown are mean ± SEM.

Infusion of VCS significantly increased striatal dihydroxyphenylacetic acid (DOPAC) levels in MPTP-treated animals (compared to animals with aCSF infusions) only at the 0.50U dose (*t* = 3.76, *P* < 0.01) (Fig 2, Table 1). Striatal DA turnover (DOPAC ⁄ DA ratio) was significantly increased in animals that received MPTP and aCSF infusions (*t* = 5.58, *P* < 0.0001) (Table 1) and was unaffected by VCS infusions in MPTP-treated animals (Table 1). Administration of VCS to normal animals had no significant effects on striatal DOPAC levels or DOPAC/DA ratio (Table 1).

### Sialidase treatment partially protects against loss of SNc DA neurons in MPTP-treated mice

**Fig 3:**
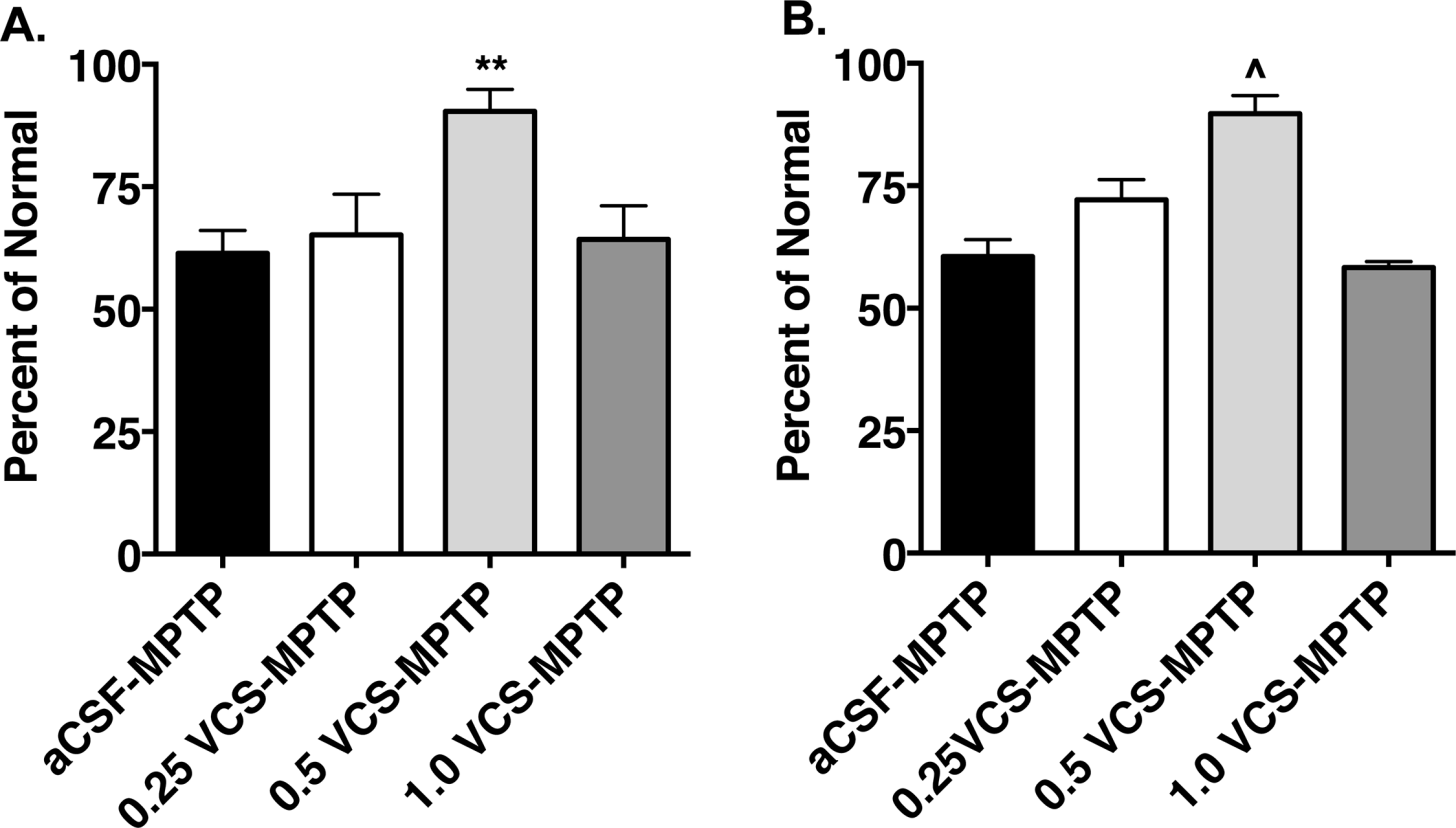
Effects of sialidase treatment on substantia nigra dopamine neurons. Sub-acute MPTP treatment (aCSF-MPTP) resulted in significant decreases in the number of tyrosine hydroxylase immunopositive (A.) and Nissl-stained (B.) cells in the substantia nigra pars compacta. Intraventricular infusion of VCS at the 0.50 U dose resulted in a significant sparing of both tyrosine hydroxylase immunopositive and Nissl-stained cells (**p<0.01 vs aCSF-MPTP; ^p<0.001 vs. aCSF-MPTP).

The number of TH^+^ cells in the SNc (counted on one side in each animal) was significantly influenced by MPTP administration and by sialidase treatment (F_(4,32)_ = 9.10, *P* < 0.0001; Table 2). MPTP administration caused a 38.7 ± 4.7% loss of SNc DA neurons, compared to normal/aCSF controls (*t* = 4.63, *P* < 0.001). MPTP-treated animals that received the 0.50U dose of VCS had a significant sparing of SNc DA neurons (9.6 ± 4.5% loss, *t* = 3.37, *P* < 0.01 vs. MPTP/aCSF) (Table 2, Fig 3), while the other doses of VCS had no significant effect on DA cell number. Similarly, the number of cresyl violet-stained cells in the SNc was significantly influenced by MPTP administration and by sialidase treatment (F_(4,26)_ = 15.73, *P* < 0.0001). MPTP administration caused a 39.4 ± 3.4% loss of cresyl violet-stained neurons, compared to normal/aCSF controls (*t* = 6.08, *P* < 0.0001). MPTP-treated animals that received the 0.50U dose of VCS showed a significant sparing of cresyl violet-stained neurons (10.3 ± 3.7% loss, *t* = 4.34, *P* < 0.001 vs. MPTP/aCSF) (Fig 3).

**Table 2:** Effects of sialidase and GM1 ganglioside administration on unilateral substantia nigra pars compacta dopamine neuron cell counts.

Treatment	*n*	TH^+^ Neurons
aCSF/Saline	8	4,092 ± 140.9
aCSF/MPTP	7	2,510 ± 192.1 ^a^
0.25U/ml VCS/MPTP	8	2,666 ± 341.5
0.50U/ml VCS/MPTP	7	3,699 ± 184.7 ^b^
1.0U/ml VCS/MPTP	7	2,630 ± 280
Normal/Saline	7	4,261 ± 182.2
MPTP/Saline	7	2,479 ± 242.0 ^c^
MPTP/GM1	8	4,063 ± 205.0 ^d^

^a^ P < 0.001 vs. aCSF/Saline

^b^ P < 0.01 vs aCSF-MPTP

^c^ P < 0.0001 vs. Normal/Saline

^d^ P < 0.0001 vs. MPTP/Saline.

Data shown are mean ± SEM.

### Sialidase treatment altered the ganglioside expression profiles

**Fig 4:**
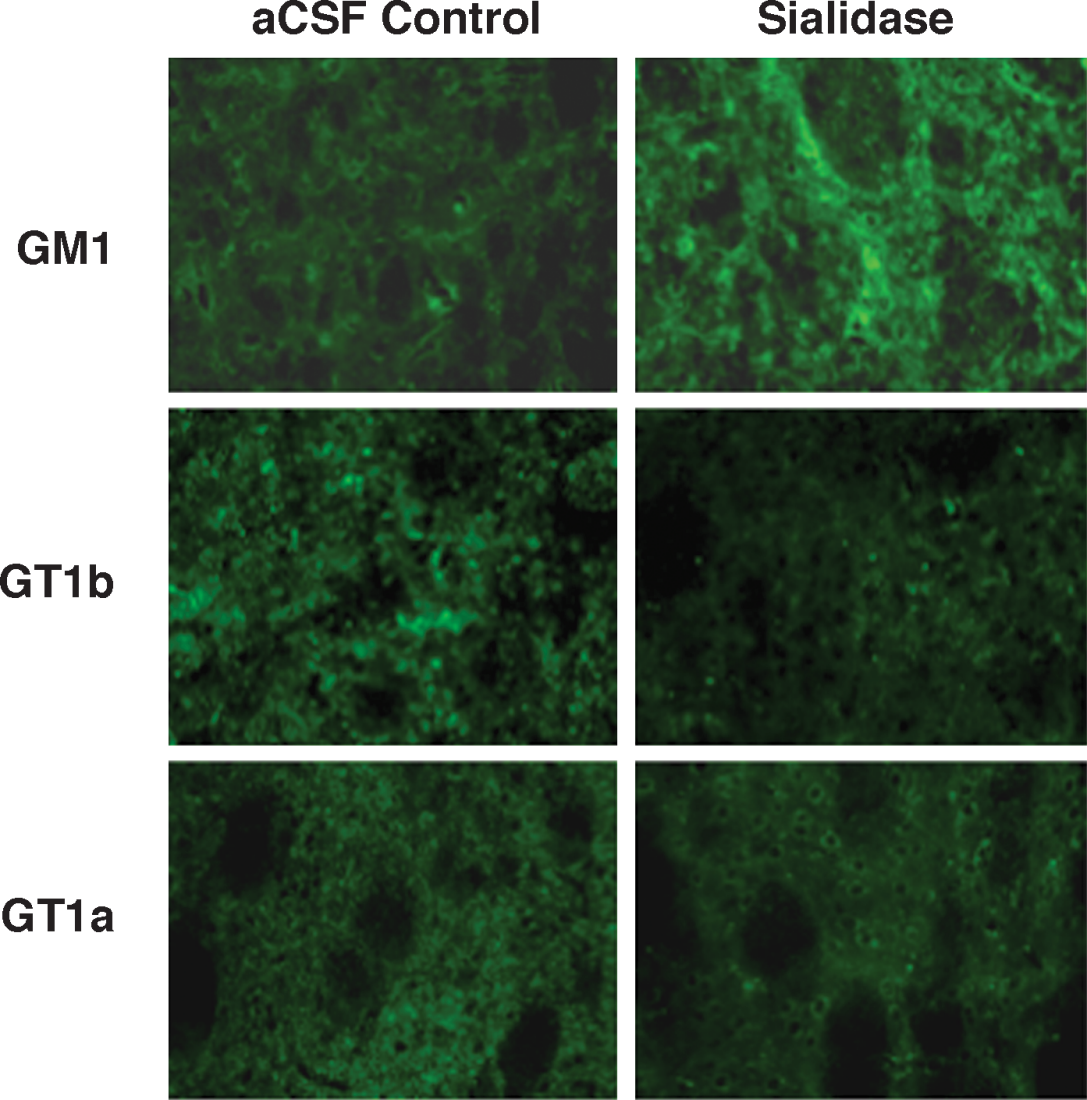
Intraventricular sialidase infusion altered ganglioside expression. In normal animals, intraventricular infusion of the 0.5U dose of *Vibrio cholera*e (VCS) sialidase resulted in increased expression of GM1 ganglioside and decreased expression of gangliosides GT1b, GD1a, in comparison to animals that received aCSF infusion only, as detected immunohistochemically in striatal sections.

**Fig 5:**
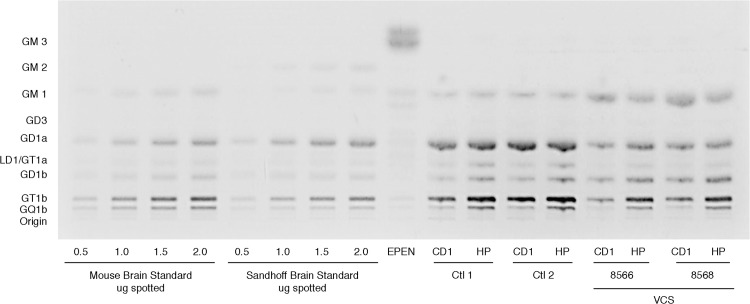
High-performance thin-layer chromatogram of mouse brain ganglioside distribution in control (Ctl 1, Ctl 2) and experimental (VCS, #8566, #8568) brain tissue. The plate was developed by one ascending run with chloroform:methanol:water(50:40:10 by vol) that contained 0.02% CaCl_2_:2H_2_O. The bands were visualized by resorcinol-HCl spray and heating at 100° C for 10 min. Note the split band for GM1 in lane 10. Both GM1 bands were combined for the data presented in Table 1. EPEN = GM3-enriched ependymoblastoma standard. Note the increase in GM1 in VCS animals and decreased GD1a, GT1a, GT1b, and GQ1b, in striatal (CD) and hippocampus (HP) samples.

VCS-treated mice showed an increase in GM1 expression and decreased expression of GT1b, GD1a, GQ1b, consistent with the expected effects of VCS on gangliosides (Fig 4). These changes in ganglioside expression were observed immunohistochemically in striatal sections and further observed by HPTLC, which further showed that GM2 and GM3 levels were unaffected by the treatment (Fig 5, Table 3). Immunohistochemistry data suggested a decrease in GD3 in VCS-treated animals, however, this could not be confirmed by HPTLC.

**Table 3:** Effects of sialidase administration on brain gangliosides.

Treatment	Region	GM3	GM2	GM1	GD1a	GD1b	GT1b	GQ1b
Control	CP	1.5 ± 0.3	1.5 ± 0.1	12.3 ± 0.8	35.9 ± 1.2	10.0 ± 0.7	22.0 ± 0.2	7.1 ± 0.2
VCS	CP	1.4 ± 0.2	1.8 ± 0.1	32.5 ± 0.4^	23.6 ± 0.6^	15.1 ± 0.4^	13.8 ± 0.2^	4.6 ± 0.9*
Control	HIPP	1.9 ± 0.2	1.2 ± 0.2	10.3 ± 0.7	29.7 ± 1.2	11.2 ± 0.8	22.6 ± 0.5	10.8 ± 0.6
VCS	HIPP	1.7 ± 0.1	1.2 ± 0.1	21.1 ± 0.2^	21.9 ± 0.4^	17.2 ± 0.7^	18.7 ± 1.3^+^	8.8 ± 0.4

^P < 0.0001 vs. Control

*P < 0.05 vs. Control

^+^P < 0.01 vs. Control

### Sialidase treatment had no effect on dopamine uptake

**Fig 6:**
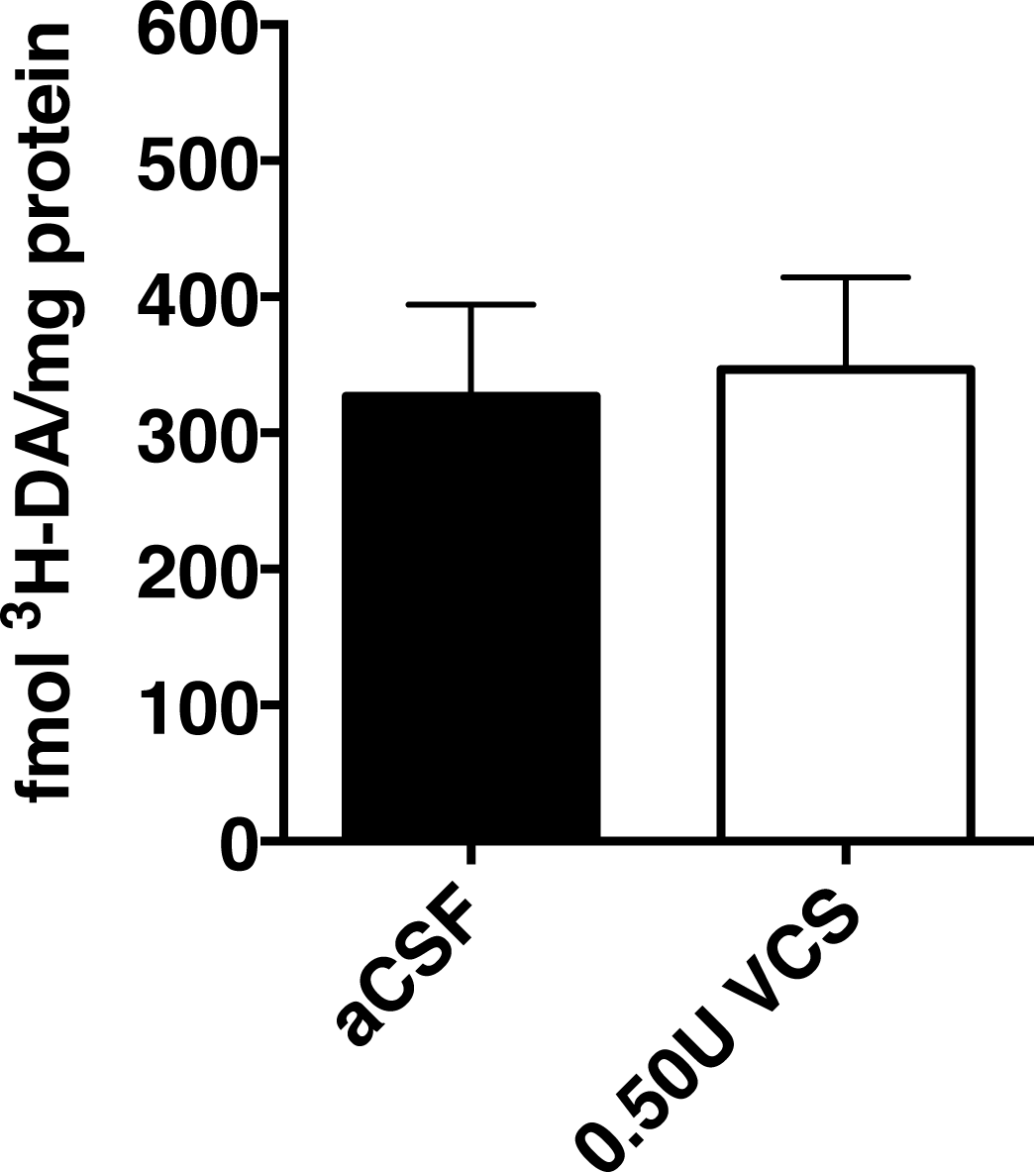
Striatal uptake of tritiated dopamine (^3^H-DA). Striatal homogenates were incubated with ^3^H-DA in the presence (non-specific) or absence (total) of the dopamine uptake inhibitor mazindol. Mazindol effectively blocked DA uptake; sialidase treatment did not. Specific uptake of DA was not altered by *Vibrio cholera*e (VCS) sialidase (0.5U/mL, infused by osmotic minipump for 12 days).

Since sialidase administration began prior to MPTP administration, we performed DA uptake studies to assess whether administration of sialidase could have altered the uptake of DA (and thus potentially MPTP), which could potentially interfere with the mechanism responsible for the toxic effect of MPTP on DA neurons [28]. Administration of VCS (0.50U dose) had no significant effect on DA uptake (Fig 6).

### Comparison of effects of GM1 and sialidase administration on MPTP-treated mice

**Fig 7:**
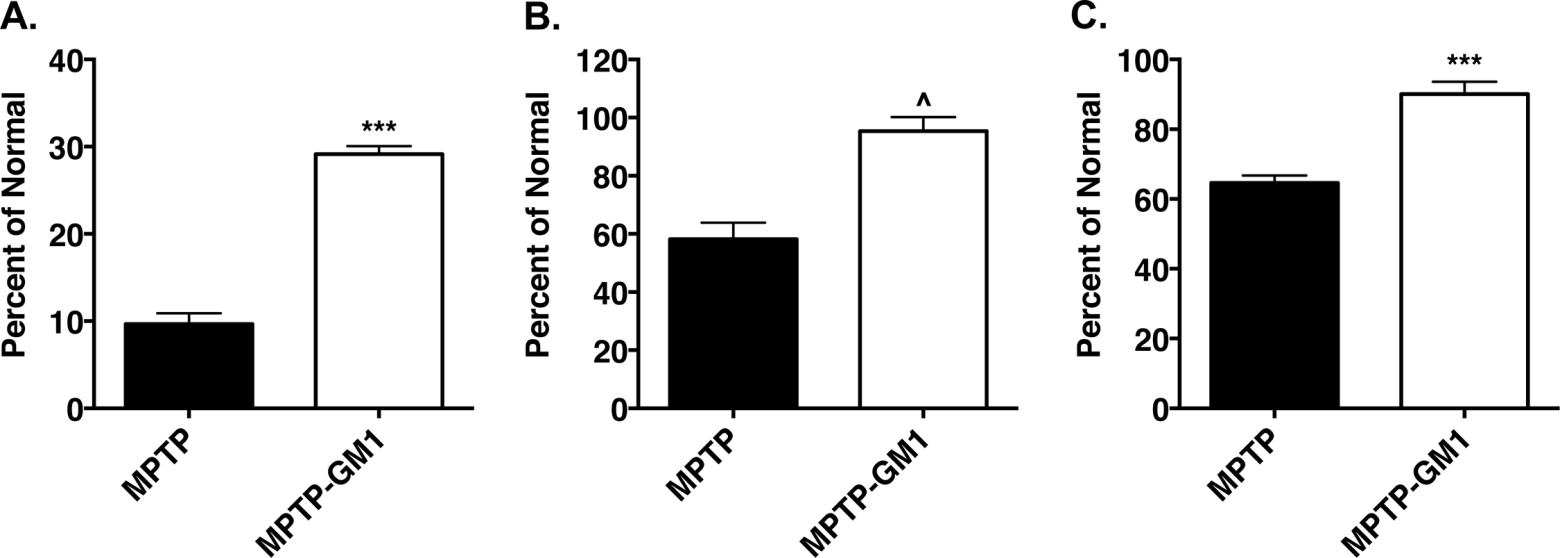
Effects of systemic administration of GM1 ganglioside (30 mg/kg, i.p.) on striatal dopamine levels and substantia nigra dopamine neurons. GM1 was administered daily for 14 days beginning 24 hours after the last MPTP injection. GM1 administration significantly increased striatal dopamine levels (A., ***p<0.001 vs. MPTP) and resulted in a significant sparing of both tyrosine hydroxylase immunopositive (B., ^p<0.0001 vs. MPTP) and Nissl-stained (C., ***p<0.001 vs. MPTP) cells in the substantia nigra pars compacta.

Consistent with prior studies [7, 29], two weeks of GM1 administration had a significant sparing effect on striatal DA levels (70.8 ± 0.9% reduction vs. 90.3 ± 1.2% reduction in MPTP/saline-treated animals, *t* = 12.61, *P* < 0.0001) (Fig 7, Table 1). DOPAC levels were also significantly affected by GM1 administration (*t* = 4.77, *P* = 0.0003 vs. MPTP/saline) and DOPAC/DA ratios were significantly decreased, compared to MPTP/saline-treated animals (*t* = 4.84, *P* = 0.0003) (Table 1). GM1 treatment also had a significant sparing effect on the number of TH^+^ cells (41.8 ± 5.7% loss in MPTP saline animals vs. 4.6 ± 4.8% loss in MPTP/GM1 animals, *t* = 5.032, *P* = 0.0002) and the number of cresyl violet-stained cells in the SNc (35.4 ± 2.1% loss in MPTP/saline animals vs. 9.9 ± 3.5% loss in MPTP/GM1 animals, *t* = 6.04, *P* < 0.0001) (Fig 7).

**Fig 8:**
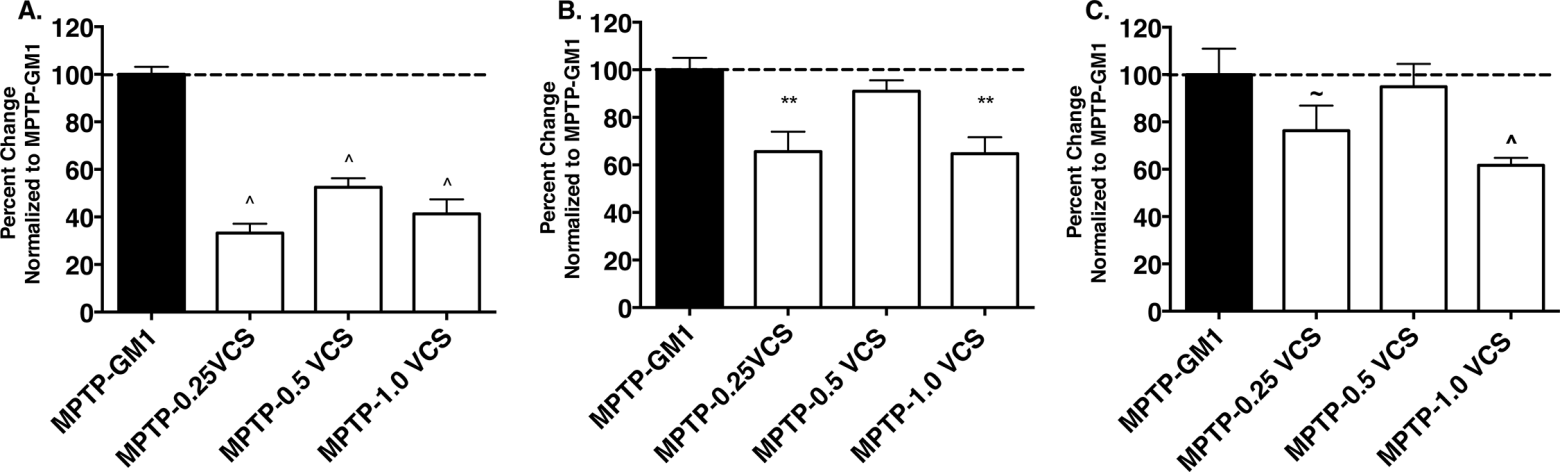
Comparison of efficacy of sialidase infusion versus systemic GM1 administration. At all doses, *Vibrio cholera*e sialidase (VCS) infusion was less effective than systemically administered GM1 at increasing striatal dopamine levels (A., ^p<0.0001 vs. MPTP-GM1). However, at the 0.5U dose, VCS was not significantly different from GM1 in the ability to spare tyrosine hydroxylase immunopositive neurons in the substantia nigra pars compacts (B.) while at a lower and higher dose, VCS was less effective than GM1 (**p<0.001 vs. GM1). Similarly, at the 0.5U dose, VCS was not significantly different from GM1 in the ability to spare Nissl-stained neurons in the substantia nigra pars compacts (C.) while at a lower and higher dose, VCS was less effective than GM1 (^~^p<0.001 vs. GM1; ^p<0.0001 vs. GM1).

In comparison with GM1 administration, VCS administration had a smaller effect on striatal DA levels in MPTP-treated animals than did GM1 administration however, at the 0.50U dose, sparing effects on the number of TH^+^ and cresyl violet-stained cells were similar to that observed with GM1 (Fig 8).

## Discussion

Administration of GM1 ganglioside has had beneficial effects on behavior, striatal DA levels and SNc DA neurons in animals with experimentally-induced Parkinsonism (ex., [3–10]. In PD patients, long-term GM1 use has shown potential for disease modification [12–14]. Although the precise mechanisms underlying the clinical benefit from systemic GM1 administration are currently unknown and are likely multi-factorial, GM1 levels appear to be decreased in the brain of patients with PD [30, 31] and systemic administration of GM1 (i.e, GM1 replacement therapy) may deliver a sufficient amount of GM1 to SN DA neurons to stabilize them and promote their survival. Recent work by Hadaczek et al. [31] suggest that GM1 may regulate glial cell-derived neurotrophic factor (GDNF) signaling, a function necessary for maintaining the health of DAergic neurons. GM1 was shown to be required for assembly of the GDNF receptor and effective GDNF signaling was dependent on an adequate level of GM1 [31]. It possible that such an activity of GM1 could contribute to its neuroprotective potential. However, given the challenges associated with sourcing GM1 and delivering it the brain, we sought an alternative therapeutic approach to increasing GM1 levels in the brain. Taking advantage of the known biochemistry of ganglioside biosynthesis and degradation, we explored the potential for enhancing GM1 levels in the brain by intracranial administration of VCS sialidase, which cleaves the glycosidic linkages between terminal sialic acids of complex gangliotetraose gangliosides GD1a, GD1b, and GT1b, and GQ1b to yield increased levels of GM1[17] and the potential for this enzyme to exert a neuroprotective effect in the MPTP mouse model of Parkinsonism.

Intracranial administration of VCS resulted in the expected changes in a- and b-series gangliosides as described previously by Dhanushkodi et al. [17]. That is, GM1 levels significantly increased while GD1a, GT1b, and GQ1b levels decreased. It is interesting that the percent distribution of GD1b was elevated rather than decreased relative to the other gangliosides. GD1b could be located in a compartment not readily accessible to VCS, which could have artificially magnified the content of GD1b. For example, GD1b is present in myelin and it is possible that with a loss of cell bodies or terminals, the appearance of gangliosides enriched in white matter could be magnified. However, it is most likely that the increase in GM1 levels were responsible for the partial sparing of nigrostriatal DA neurons following MPTP administration, as the DA neuron-sparing effect from VCS administration was similar to that obtained with systemic GM1 administration. Considering the similar efficacy of VCS and GM1 administration on SNc DA neurons, the finding that VCS administration did not have as robust an effect on striatal DA levels as did GM1 administration was surprising and the reasons for this are not entirely clear. It is possible that the decrease in GD1a and several b-series gangliosides may have affected dopamine synthesis. Although there are few studies of the effects of gangliosides other than GM1 on animal models of PD, one study did show that stereotaxic injection of GD1a into the striatum of MPTP-treated mice significantly increased striatal DA levels [32]. It is possible that both GM1 and GD1a contribute to the recovery of DA synthesis in MPTP-lesioned animals; in the current study, there was a significant decrease in GD1a in VCS-treated animals. However, considering the degree of sparing of SNc DA neurons, it is likely that striatal DA would recover and with longer post-MPTP survival times we would expect to see recovery of striatal DA levels at least to the extent seen with GM1 administration.

The neuroprotective effects associated with VCS administration were likely due to the enhancement of endogenous GM1 levels and not alterations in the kinetics of MPTP-mediated toxicity. MPP^+^ toxicity is dependent upon the uptake of MPTP into DAergic neurons via the dopamine transporter (DAT) [33] and DAT is a glycoprotein containing N-linked sugars and sialic acids [34, 35]. It was previously suggested that DA uptake was inhibited in a non-competitive way by treatment of synaptosomes with high concentrations of neuraminidase (sialidase) from *Vibrio cholerae* [36]. However, our results suggest that the function of DAT was not altered by VCS administration. Dopamine uptake rates in striatal homogenates were not altered by sialidase treatment and thus it is unlikely that sialidase administration affected the toxicokinetics of MPTP. There are no reported or predicted effects of sialidase treatment on monoamine oxidase B activity and thus, VCS-related inhibition of monoamine oxidase B activity and inhibition of conversion of MPTP to MPP^+^ is an unlikely mechanism contributing to our results.

While the current study employed a pre-treatment paradigm in order to have GM1 levels increased prior to MPTP exposure, we believe that up-regulation of endogenous GM1 levels may represent an effective alternative to systemic GM1 treatment. As stated previously, PD is a progressive neurodegenerative disorder and SNc DA neurons continue to become damaged and die throughout the course of the disease. Therefore, even with the currently used pre-treatment paradigm, one could predict that enhancing GM1 levels beginning at any point in the disease process could have potentially positive effects for patients with PD. Further studies are needed to examine the long-term effects of sialidase treatment as well as alternative strategies to increase GM1 *in vivo* in order to translate these findings into a viable treatment for PD patients.

## Conclusions

In summary, the present study found that the intraventricular administration of VCS mediated enzymatic conversion of polysialogangliosides into GM1, and in a mouse PD model, this had neuroprotective efficacy and spared SNc DA neurons to a similar extent as did systemic administration of GM1 ganglioside. Overall, the results suggest that enzymatic conversion of polysialogangliosides to GM1 *in vivo* may be a viable alternative treatment strategy for increasing GM1 levels in the brain and exerting a neuroprotective/neurorestorative effect on the damaged nigrostriatal DA system.
